# Protective Effect of an Antibody against Specific Extracellular Domain of TLR2 on Agonists-Driven Inflammatory and Allergic Response

**DOI:** 10.1155/2016/9803846

**Published:** 2016-04-24

**Authors:** Tianwu Guo, Jun Cai, Yanxia Peng, Lifang Zhang, Qiaofen Lan, Yanwen Chen, Huanjin Liao, Tong Xie, Ping Wu, Qingjun Pan

**Affiliations:** ^1^Department of Burns and Plastics, The People's Hospital of Anyang City, Anyang, Henan 455000, China; ^2^The Institute of Nephrology, Affiliated Hospital of Guangdong Medical College, Zhanjiang, Guangdong 524001, China

## Abstract

Specific blocking strategies of TLR2-mediated inflammatory signaling and hypersensitivity reactions may offer novel therapeutic strategies to prevent a variety of diseases. In this study, we investigated the blocking effects of a new anti-TLR2 antibody anti-T20 against a 20 mer peptide T20 located in the extracellular specific domain of mouse TLR2. In addition, the effects of the anti-T20* in vitro*, measuring the inhibition of the IL-6 and TNF-*α* production in response to PGN, LTA, and Pam3CSK4-stimulated RAW264.7 cells, were determined.* In vivo*, the effects of anti-T20 on a lethal anaphylaxis model using PGN-challenged OVA allergic mice, including the rectal temperature and mortality, and serum levels of TNF-*α*, IL-6, and LTC4 were assayed. The results showed that anti-T20 specifically bound to TLR2 and significantly inhibited PGN, LTA, and Pam3CSK4-driven TNF-*α* and IL-6 production by RAW264.7 cells. Also, anti-T20 protected OVA allergic mice from PGN-induced lethal anaphylaxis, and the serum levels of TNF-*α*, IL-6, and LTC4 of anti-T20 treated PGN-challenged OVA allergic mice were decreased as compared to isotype control of anti-T20 treated mice. In summary, this study produced a new antibody against the specific extracellular domain of TLR2 which has protective effect on TLR2 agonists-driven inflammatory and allergic response.

## 1. Introduction

Toll-like receptors (TLR) are known to be major actors of the innate response, most of them principally recognize pathogen-associated molecular pattern molecules (PAMPs), and are able to transduce signals through different pathways. These signals are the first step towards cell pathway activation and towards a more specific response. One of the important TLRs, TLR2, is widely distributed on the surface of monocyte-macrophages, dendritic cells, mast cells, basophils, and other cells [1–4] and can recognize various types of ligands including peptidoglycan (PGN), lipoteichoic acid (LTA), Pam3CSK4, zymosan, HSP60, hyaluronic acid, the apoptosis nucleosome, and high mobility group box protein 1 (HMGB1) [5–7].

Similar properties of cell-mediated signal transduction activity and analogous inflammatory cytokine release following ligands interaction with TLR2 have been seen. Agonists of TLR2 can generate both antimicrobial and antitumorigenic effects with subsequent benefits in terms of protective host immunity, while they also can induce hyperinflammatory and hypersensitivity reactions, which have the potential to promote detrimental effects on human health [8, 9]. Accordingly, specific blocking strategies of TLR2 may offer novel therapeutic strategies to prevent a variety of diseases.

In this study, we produced a new anti-TLR2 antibody and investigated its blocking effects on agonists-stimulated mouse macrophage cell line RAW264.7* in vitro* and on OVA-specific allergic mice from PGN-induced lethal anaphylaxis, which provides a novel strategy to prevent TLR2 agonist-mediated inflammation and promotion of allergic immune responses.

## 2. Materials and Methods

### 2.1. Animals and Raw264.7

New Zealand white rabbits and BALB/c mice were obtained from the Experimental Animal Center of Southern Medical University (Guangzhou, China) and the Experimental Animal Center of Guangdong Medical College (Zhanjiang, China). The animals were maintained at 25°C in a 12 h equal light : dark cycle with 50% humidity and were fed with commercial feed and sterile water* ad libitum*. The animal experiments were approved by the Ethics Committee for Experimental Animals at Guangdong Medical College and were performed according to relevant national and international guidelines. In all experiments, efforts were made to minimize suffering, and any animals appearing moribund during the course of experimentation were immediately anesthetized with pentobarbital sodium (100–150 mg/kg, i.p.). RAW264.7 cells were obtained from the Tissue Type Culture Collection of the Chinese Academy of Sciences (Shanghai, China).

### 2.2. Reagents and Instruments

Ovalbumin (OVA), bovine serum albumin (BSA), and Freund's adjuvant were obtained from Sigma-Aldrich Chemical Co. (St. Louis, MO, USA). Blue-carrier protein (BC), carbodiimide (EDC), the BCA kit, and anti-mouse IgE-HRP were obtained from Pierce Biotechnology (Rockford, IL, USA). Sepharose 4B was obtained from Amersham Pharmacia Biotech (Piscataway, NJ, USA), and commercialized mouse TLR2 antibody (T2.5) was obtained from BioLegend (San Diego, CA, USA). PGN, LTA, and Pam3CSK4 were obtained from InvivoGen (San Diego, CA, USA). Irrelevant rabbit control antibody, anti-mouse IgG, IgE-HRP, FITC-goat anti-mouse and FITC-goat anti-rabbit, TNF-*α*, and IL-6 ELSIA kits were all obtained from Wuhan Boster Biological Technology, Ltd. (Wuhan, China). Leukotriene C4 (LTC4) EIA kits were obtained from Cayman Chemical Company (Ann Arbor, MI, USA).

### 2.3. Design and Synthesis of Antigenic Peptides

A 20 mer peptide (named as T20) encoding the amino acid sequence (DSQS LKSI RDIH HLTL HLSE) on the basis of the predicted B cell dominant epitope of mouse TLR-2 was synthesized by Hybio Pharmaceutical Company (Shenzhen, China) [10]. Predicted Antigenic Peptide software provided by the Harvard University Molecular Immunology Foundation (website: http://imed.med.ucm.es/Tools/antigenic.pl) was used to predict epitopes in this sequence.

### 2.4. Preparation of the Immunogen and Animal Immunization

In the presence of EDC, T20 was cross-linked with BC, BSA, and rabbit IgG, respectively, to obtain the cross-linked T20-BC, T20-BSA, and the rabbit IgG-T20 (rIgG-T20) by conventional methods. T20-BC acted as an immunogen, and two New Zealand white rabbits were immunized six times with this immunogen, at a dose of 1 mg each time by conventional immunizing methods.

### 2.5. Anti-T20 Purification and Identification

Anti-T20 was purification with a cross-linked product Sepharose 4B-rIgG-T20 column. RAW264.7 cells were cultured and collected by trypsin digestion and then detected by flow cytometry and western blot. 1 × 10^6^ RAW264.7 cells were treated with anti-T20 (1 *μ*g/test), or anti-T20 (1 *μ*g/test) plus T20 (1 *μ*g/test), or T2.5 (1 *μ*g/test) at room temperature for 30 min. Irrelevant rabbit antibody was used as a control and washed three times with PBST containing 1% BSA, at 350 g for 5 min by centrifugation, following which FITC-goat anti-mouse antibody or FITC-goat anti-rabbit antibody was added, and incubated in the dark at room temperature for 30 min, washed twice, and then resuspended in 200 *μ*L PBST containing 1% BSA and detected by flow cytometry. The expression of TLR2 protein in RAW264.7 cells was detected by anti-T20 (1 *μ*g/mL), or anti-T20 (1 *μ*g/mL) plus T20 (1 *μ*g/mL), or T2.5 (1 *μ*g/mL) using western blot routinely with *β*-actin as internal reference. The BCA kit was used for quantitative protein measurements of the collected samples.

### 2.6. Effects of Anti-T20 on PGN, LTA, and Pam3CSK4-Challenged RAW264.7 Cells

Approximately 10^6^ RAW264.7cells/well were seeded into six-well plates, in IMDM medium containing 5% bovine serum, at 37°C in 5% CO_2_ incubator, and grouped by PGN (1 *μ*g/mL), PGN (1 *μ*g/mL) plus anti-T20 antibody (1 *μ*g/mL), PGN (1 *μ*g/mL) plus anti-T20 antibody (5 *μ*g/mL), and PGN (1 *μ*g/mL) plus anti-T20 antibody (25 *μ*g/mL), or LTA (0.1 *μ*g/mL), LTA (0.1 *μ*g/mL) plus anti-T20 antibody (0.1 *μ*g/mL), LTA (0.1 *μ*g/mL) plus anti-T20 antibody (0.5 *μ*g/mL), and LTA (0.1 *μ*g/mL) plus anti-T20 antibody (2.5 *μ*g/mL); Pam3CSK4 (0.1 *μ*g/mL), Pam3CSK4 (0.1 *μ*g/mL) plus anti-T20 antibody (0.1 *μ*g/mL), Pam3CSK4 (0.1 *μ*g/mL) plus anti-T20 antibody (0.5 *μ*g/mL), and Pam3CSK4 (0.1 *μ*g/mL) plus anti-T20 antibody (2.5 *μ*g/mL) following culture, and at 6 h and 12 h time-points, supernatants were collected, respectively, from each of the groups and frozen at −40°C until detected by commercial ELISA kits.

### 2.7. Effects of Anti-T20 on PGN-Challenged OVA Allergic Mice

BALB/c mice of six-week-old BALB/c mice were multipoint immunized in the abdominal subcutaneous areas with OVA (100 *μ*g) emulsified with AL(OH)_3_ on days 0, 10, and 20. The anti-OVA IgG and IgE antibody levels were detected on day 21. The dose of OVA was intravenously administered at 30 mg/Kg.

Then, the OVA allergic mouse model was established and divided into the following groups: Group 1, the OVA (30 mg/Kg) challenged group; Group 2, the OVA (30 mg/Kg) plus PGN (100 *μ*g/mouse) experimental group; Group 3, the OVA (30 mg/Kg) plus PGN (100 *μ*g/mouse) plus anti-T20 antibody (100 *μ*g/mouse) group, which was the intervention experimental group; Group 4, the OVA (30 mg/Kg) plus anti-T20 antibody (100 *μ*g/mouse) group; and Group 5, the isotype negative control group of OVA (30 mg/Kg) plus PGN (100 *μ*g/mouse) plus rabbit irrelevant antibody (100 *μ*g/mouse), and also normal mice as control group. Changes in rectal temperature and mortality were determined, respectively.

At 30 min and 60 min after OVA challenge and other treatments, serum was isolated by centrifugation of blood samples and frozen at −40°C until TNF-*α*, IL-6, and LTC4 analysis.

### 2.8. Statistical Analysis

Values were expressed as mean ± SD. Using the SPSS Kaplan-Meier 13.0 survival curve program, animal survival was compared for statistically significant differences using the Breslow-Gehan-Wilcoxon test. Comparisons between groups were done using the unpaired Student *t* test. *p* < 0.05 was considered statistically significant.

## 3. Results

### 3.1. T20 Peptide Synthesis and Antigenic Predictive

**Figure 1:**
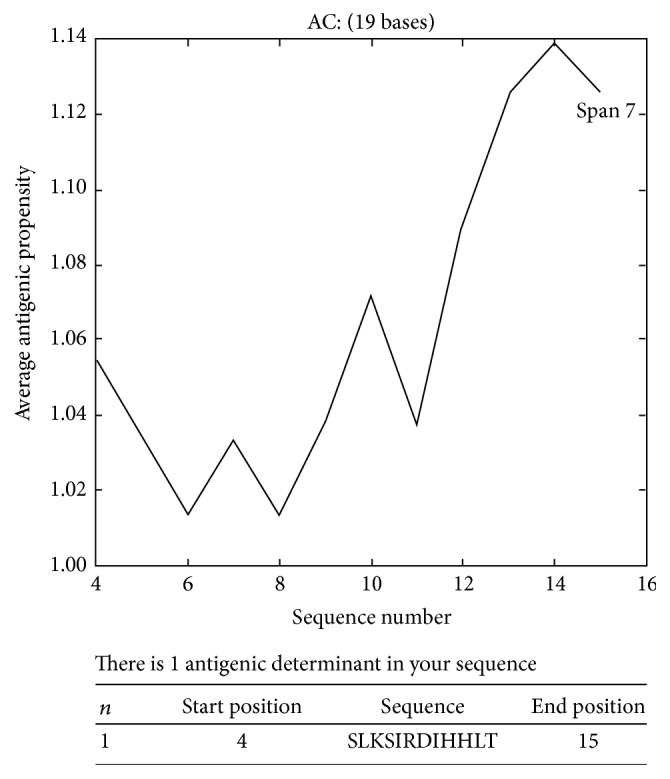
Predicted antigenic determinants in a 20 mer peptide (designed as T20) located in the extracellular specific domain of mouse TLR2. Predicted Antigenic Peptide software provided by the Harvard University Molecular Immunology Foundation (website: http://imed.med.ucm.es/Tools/antigenic.pl) was used to predict epitopes in this sequence.

The T20 encoding the amino acid sequence DSQS LKSI RDIH HLTL HLSE contained only a single antigenic determinant (Figure 1).

### 3.2. Identification of Anti-T20 Antibody

**Figure 2:**
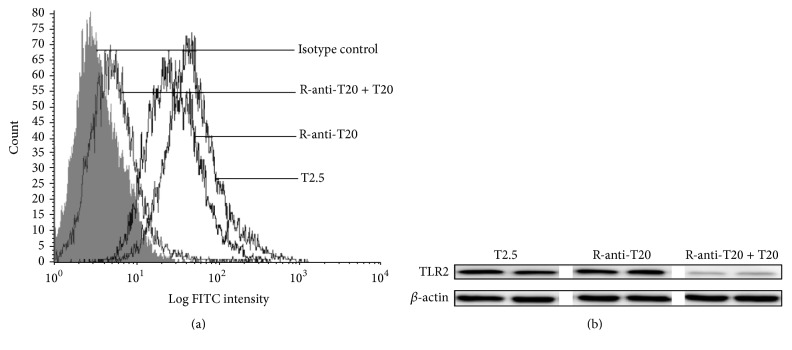
Detection of TLR2 expression by RAW264.7 cells with anti-T20. The expression of TLR2 by RAW264.7 cells was detected by (a) flow cytometry and (b) western blot with anti-T20, or anti-T20 plus T20, or T2.5 as described in Section 2.

Approximately 80 mL serum was collected from the immunized rabbits with BC-T20. T20-BSA conjugates were used as a coating antigen (10 *μ*g/mL), and anti-T20 IgG was approximately a titer of 1 : 100. Anti-T20 (12.8 mg in a total volume of 2 mL) was purified with Sepharose 4B-rIgG-T20 affinity column. Flow cytometry showed that rabbit anti-T20 (r-anti-T20) could bind to RAW264.7 cells and that this combination could be blocked by T20 peptide. Fluorescence microscopy also showed that anti-T20 (r-anti-T20) could bind to RAW264.7 cells (Figure 2).

### 3.3. Inhibition of Anti-T20 on PGN, LTA, and Pam3CSK4-Driven TNF-*α* and IL-6 Secretion by RAW264.7 Cells

**Figure 3:**
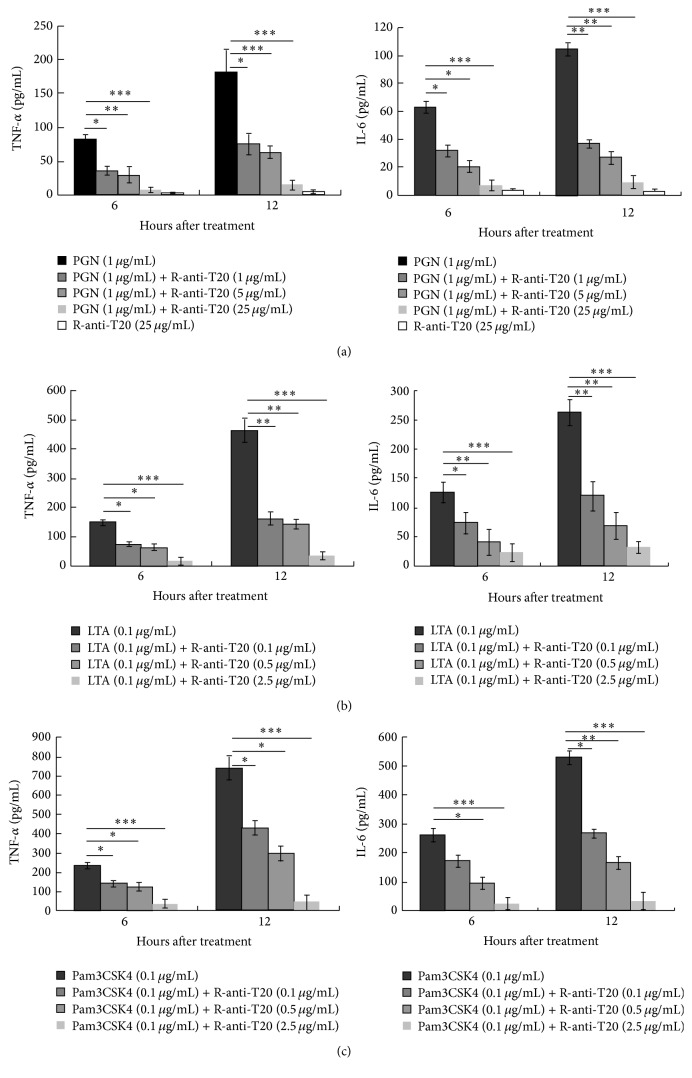
Inhibition of anti-T20 on PGN, LTA and Pam3CSK4-driven TNF-*α* and IL-6 secretion by RAW264.7 cells. The production of TNF-*α* and IL-6 by RAW264.7 cells stimulated with (a) PGN, (b) LTA, and (c) Pam3CSK4 in the presence or absence of anti-T20. Supernatants were collected separately from each of the respective groups, and detecting TNF-*α* and IL-6 levels by commercial ELISA kits as described in Section 2. Data are the mean ± SD of triplicates from an experiment that was repeated three times with similar results. ^*∗*^ *p* < 0.05, ^*∗∗*^ *p* < 0.01, and ^*∗∗∗*^ *p* < 0.001 versus control.

RAW264.7 cells stimulated with PGN and LTA and Pam3CSK4 produced large quantity of TNF-*α* and IL-6 at 6 h and 12 h, which can be significantly inhibited by anti-T20 by a dose-dependent manner (Figure 3).

### 3.4. Protective Effect of Anti-T20 on PGN-Challenged OVA Allergic Mice

OVA-specific IgG titers were approximately 1 : 500,000 and IgE titers were approximately 1 : 400 detected by ELISA method using purified OVA as the coating antigen (10 *μ*g/mL).

**Figure 4:**
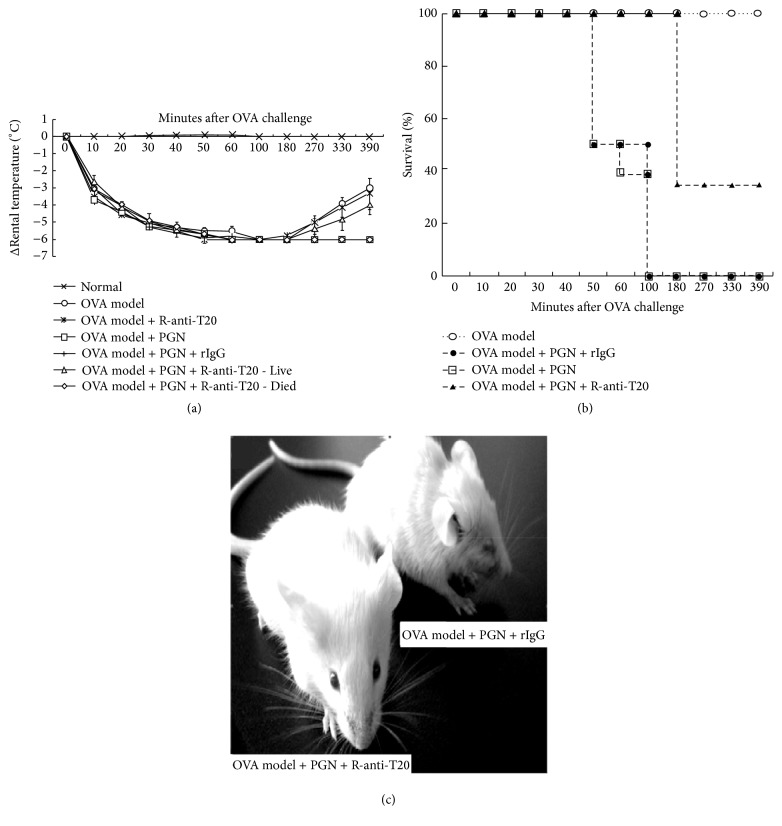
Effects of anti-T20 on PGN- challenged OVA allergic mice* in vivo*. (a) The rectal temperature, (b) the survival rate, and (c) the outward appearance of OVA allergic mice (OVA model), OVA allergic plus r-anti-T20 mice (OVA model + anti-T20), OVA allergic plus PGN challenge mice (OVA model + PGN), OVA allergic plus PGN challenge plus rabbit-anti-T20 mice (OVA model + PGN + r-anti-T20), OVA allergic plus PGN challenge plus rabbit IgG isotype control mice (OVA model + PGN + rIgG), and normal mice as described in Section 2 (*n* = 12). Changes in rectal temperature and mortality were determined, respectively.

**Figure 5:**
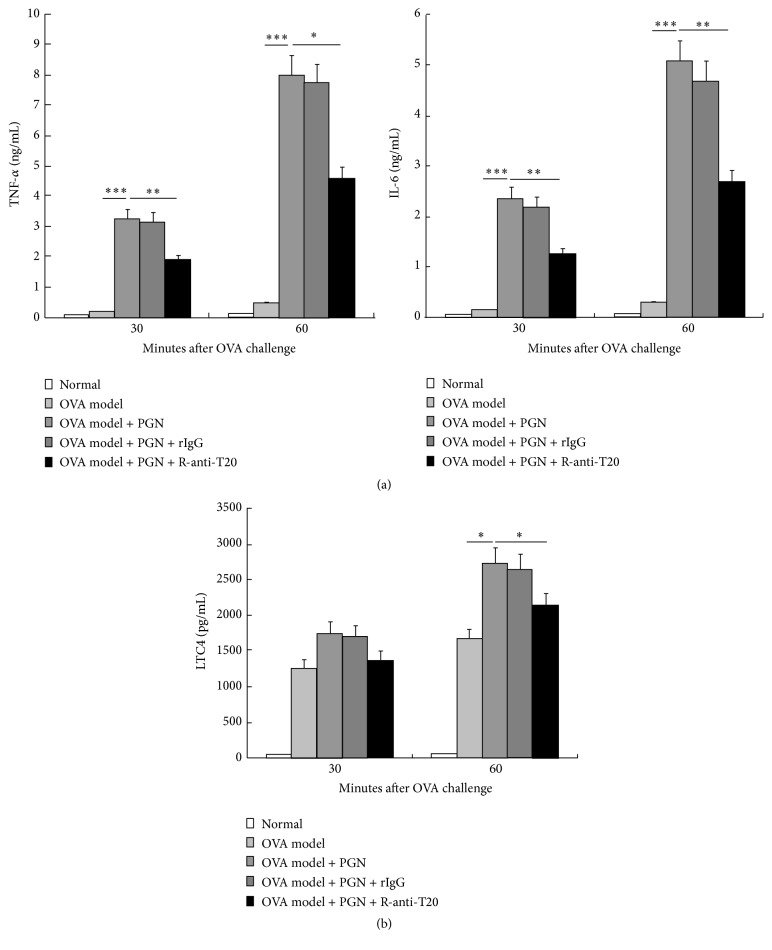
Effects of anti-T20 on serum levels of TNF-*α*, IL-6, and LTC4 of PGN-challenged OVA allergic mice. The serum levels of (a) TNF-*α*, IL-6, and (b) LTC4 of OVA allergic mice (OVA model), OVA allergic plus PGN challenge mice (OVA model + PGN), OVA allergic plus PGN challenge plus anti-T20 mice (OVA model + PGN + r-anti-T20), OVA allergic plus PGN challenge plus rabbit IgG isotype control mice (OVA model + PGN + rIgG), and normal mice, as described in Section 2 (*n* = 6). Changes in rectal temperature and mortality were determined, respectively. ^*∗*^ *p* < 0.05, ^*∗∗*^ *p* < 0.01, and ^*∗∗∗*^ *p* < 0.001 versus control.

OVA allergic mice showed a typical allergic reaction but not lethal anaphylaxis (Figure 4). The rectal temperature reached its lowest point after OVA challenge at 60 min, and lasted for about 120 min, and then gradually returned to basal temperature (Figure 4(a)). PGN-challenged OVA allergic mice (OVA model plus PGN) exhibited a 100% mortality rate within 100 min (Figure 4(b)), and the rectal temperature achieved its lowest point after OVA challenge at 60 min and did not recover to basal temperature (Figure 4(a)). Anti-T20 (OVA model plus PGN plus r-anti-T20) significantly protected OVA-specific allergic mice from PGN-induced lethal anaphylaxis (Figures 4(a) and 5), and the protective rate was 33.3% (Figure 4(b)). The rectal temperature of living mice also gradually returned to basal temperatures (Figure 4(a)), while the anti-T20 isotype control on OVA model plus PGN (OVA model plus PGN plus rIgG) and anti-T20 on OVA model (OVA model plus anti-T20) displayed no such protective effect (Figure 4). The outward appearance of mouse treated with OVA plus PGN plus rIgG (OVA model plus PGN plus rIgG) and mouse treated with OVA plus PGN plus r-anti-T20 (OVA model plus PGN plus r-anti-T20) was shown in Figure 4(c).

We also investigated the effects of these treatments on serum levels of TNF-*α*, IL-6, and LTC4 in OVA allergic mice. The results showed that serum levels of TNF-*α* and IL-6 in PGN- challenged OVA allergic mice (OVA model plus PGN) were significantly increased at 30 min and 60 min after OVA challenge as compared with OVA allergic mice (OVA model), and anti-T20 treatment (OVA model plus PGN plus r-anti-T20) markedly attenuated this increase, but not isotype control of anti-T20 (OVA model plus PGN plus rIgG) (Figure 5(a)). Also, serum levels of LTC4 had similar tendencies, but only at 60 min after OVA challenge (Figure 5(b)).

## 4. Discussion

TLR2-mediated inflammatory signaling and hypersensitivity reactions may be blocked by at least two ways: first, the intracellular domain is prone to gene mutation or deletion or by blocking the intracellular signal transduction pathway. However, although this does not affect the extracellular segment recognition [11, 12] and combines with ligands, its application as an intervention target is limited; second, it also can be blocked by changing or interfering with the TLR2 extracellular domain, especially the recognition domain of agonists. Fujita et al. found that the TLR2 extracellular segment Ser40-Ile64, which is missing or L107E, L112E, and L115E point mutations can affect TLR2 recognition of PGN, saliva or lipopeptide mycoplasmal lipoprotein [13]; Vasselon et al. found that TLR2 could directly identify synthetic bacterial lipopeptide (sBLP), for which the extracellular LRR domain is required [14]. The structural basis of TLR2 mediated recognition of its agonists is the extracellular domain [15].

Currently, the study of TLR2-mediated identification of its agonists still cannot show the exact role of the different domains of the TLR2 extracellular domain in the ligand recognition process. In addition to the polyclonal antibody of the TLR2 extracellular domain targeted against the 26 peptide (179L-204I), other commercial and laboratory prepared anti-TLR2 monoclonal antibody preparations and polyclonal antibody against the TLR2 extracellular domain remain mostly unclear. In this study, we used a protein epitope prediction system and synthetic peptide technology that helped to predict B cell dominant epitope of mouse TLR-2, which can clearly show the target domain and avoid using the full-length and extracellular domain of mouse TLR-2 as an immunogen.

For TLR2 agonists, we used three kinds of them (i.e., Pam3CSK4 [16], LTA [17, 18], and PGN [19, 20]) to stimulate RAW264.7 cells and found that anti-T20 could inhibit these three agonist-mediated inflammation and driven allergic responses* in vitro* and* in vivo*.

In view of gradually rising incidences of allergic diseases that are often associated with an infection, which can exacerbate allergic reaction [21], here, we found that serum levels of TNF-*α* and IL-6 in PGN-challenged OVA allergic mice were significantly increased as compared with only OVA allergic mice, but anti-T20 treatment markedly attenuated their increase. The results also showed that anti-T20 only reduces PGN/OVA mediated anaphylaxis but has no effect on only OVA induced anaphylaxis. Also, serum levels of LTC4 had similar tendencies. We noted that the role of the TLR2 signaling pathway in anaphylaxis and its presence are still controversial, and this might be related with the usage of the type and dose of the specific TLR2 agonist or antagonist, the target cell type, and the type of inflammatory factors, as well as the use of different allergic animal model [22–29]. McCurdy et al. found that* S. aureus*-derived PGN causes mast cells to release TNF-*α*, IL-4, IL-5, and IL-6, degranulation and to open Ca^2+^ channels by binding to TLR2 on the surface of mast cells [1]. Moreover, intradermal injection of PGN augments vasodilation and expansion of TLR2-dependent mast cell activation and the inflammatory response [1]. Meanwhile, the synthetic ligands Pam3Cys and PGN as adjuvants were capable of inducing a significant immune response of the Th2 type of anaphylaxis, resulting in mast cell degranulation, and inflammatory conditions that could exacerbate experimental asthma [2, 30–34]. Further, PGN binds to TLR2 of basophils to selectively induce the release of IL-4 and IL-13, but not histamine and LTC4, and also augments anti-IgE antibody-induced histamine production and the release of LTC4 [4].

In summary, this study produced a new antibody against the specific extracellular domain of TLR2 which has protective effect on TLR2 agonists-driven inflammatory and allergic response.

## Abbreviations

TLR: Toll-like receptor
ECD: Extracellular domain
PGN: Peptidoglycan
LPS: Lipopolysaccharide
LTA: Lipoteichoic acid
Pam3CSK4: Pam3CysSerLys4
HMGB1: High mobility group box protein 1
T20: 20 mer peptide containing one epitope
LTC4: Leukotriene C4.
